# Effects of exercise conducted prior to phenylketonuria-type meal on appetite, satiety hormones and energy expenditure: a randomised cross-over trial

**DOI:** 10.1038/s41430-025-01629-7

**Published:** 2025-05-23

**Authors:** Nouf A. Alghamdi, James L. Dorling, Abdulrahman Alreshidi, Konstantinos Gerasimidis, Dalia Malkova

**Affiliations:** 1 Community Health Sciences, College of Applied Medical Sciences, King Saud University, Riyadh, Saudi Arabia; 2 Human Nutrition, School of Medicine, Dentistry and Nursing, College of Medicine, Veterinary and Life Sciences, University of Glasgow, Glasgow, UK

**Keywords:** Obesity, Nutrition

## Abstract

**Background/Objectives:**

Individuals with phenylketonuria (PKU) are at increased risk for obesity, possibly due to reduced satiety induced by a PKU diet that is low protein and high carbohydrate. It is unclear how exercise alters postprandial satiety after a PKU-like meal. The objective was to examine changes in postprandial satiety, satiety hormone concentrations, energy expenditure and substrate oxidation in response to acute treadmill exercise following a PKU-like meal.

**Subjects/Methods:**

Sixteen males (mean age [±SD]: 26.5 ± 4.8 years; BMI: 23.7 ± 3.2 kg/m^2^) participated in a randomized cross-over trial with two conditions: exercise and control. Both trials involved consuming a PKU-like meal comprising naturally low-protein foods, a special low-protein food and a protein substitute. In the exercise trial, participants exercised at 60% of maximal oxygen uptake for 1 h before the meal; in the control trial, they rested. Satiety agents (peptide YY [PYY], glucagon-like peptide-1 [GLP-1] and growth differentiation factor-15 [GDF-15]), appetite, energy expenditure, fat oxidation and carbohydrate oxidation were measured.

**Results:**

Mean (±SE) appetite and postprandial PYY and GLP-1 concentrations were unaffected by exercise (*P* ≥ 0.279). However, GDF-15 was higher in the exercise trial (control: 288 ± 25 pg/mL vs. exercise: 322 ± 24 pg/mL; *P* = 0.002). Exercise increased fat oxidation (*P* = 0.013) and decreased carbohydrate oxidation post-meal (*P* = 0.022), with concomitantly lower RER (*P* = 0.005). Energy expenditure rose during exercise (*P* < 0.001), but no difference occurred postprandially (*P* = 0.543).

**Conclusions:**

Acute exercise prior to a PKU-like meal does not affect postprandial GLP-1 and PYY concentrations compared to control but GDF-15 was increased and RER was reduced, potentially improving appetite regulation.

## Introduction

Phenylketonuria (PKU) results from deficient or dysfunctional phenylalanine (an essential amino acid) hydroxylase, causing abnormal phenylalanine accumulation in the blood and brain [1]. Untreated PKU leads to intellectual disability, depression and neurological symptoms [1]. Treatment includes special low-protein foods (SLPF), protein substitutes and minimal protein intake [2–4]. Although food intake recommendations align with healthy individuals, PKU diets are lower in protein and higher in low-fibre carbohydrates (CHO) [5, 6]. This could increase obesity risk [7], given that the prevalence rate of obesity is 5.35% in PKU patients compared to 2.25% in a control sample [8].

The obesity risk in PKU suggests appetite regulation strategies may help. Lower protein [9] and higher CHO intake [10] during PKU diets may reduce satiety due to attenuated postprandial responses of the gut-derived peptide YY (PYY) and glucagon-like peptide 1 (GLP-1). Fat oxidation, which is negatively associated with food intake [11] and percentage body fat [12–14], is also lower after a PKU-like meal [15], suggesting this may be a target for improved appetite and weight management.

In healthy individuals, studies suggest that acute exercise increases postprandial GLP-1 and PYY, and reduces appetite compared to control [16, 17]. Evidence also suggests exercise, relative to control, elevates concentrations of growth differentiation factor-15 (GDF-15), which is (at odds with PYY and GLP-1) a myokine involved in weight regulation [18], given it suppresses appetite via the hindbrain [19, 20]. Additionally, exercise before [21] or after [22] meals increases fat oxidation, potentially improving appetite regulation. Thus, pre-meal exercise may enhance satiety hormone responses and fat oxidation after PKU meals, though this is untested. The aim of this study was to investigate the impact of a single exercise session conducted prior to PKU-type meal on postprandial PYY, GLP-1, GDF-15, appetite and fat oxidation in a healthy population without obesity.

## Materials and methods

### Participants

Healthy men without obesity (body mass index [BMI] between 19 and 29 kg/m^2^) and aged between 22 and 35 years were recruited. Study participants were non-smokers, had a stable body weight (<0.5 kg change in weight) for 1 month prior to enrolment (self-report), and were not on a special diet or taking medications. Exclusion criteria included a chronic illness (e.g. cardiovascular disease, diabetes, cancer, hypertension), an eating disorder, allergies to study foods and a history of gastrointestinal operations. The present study was a proof of concept, mechanistic study. Hence, because of this and the low incidence of the disorder in the UK (1 in 10,000) [23], patients with PKU were not recruited.

All participants gave written informed consent prior to participation. The Research Ethics Committee at the University of Glasgow approved the study and the study was performed in accordance with the Declaration of Helsinki. The study was registered at clinical trials.gov: NCT04302285.

### Study design overview

The study applied a randomised, crossover design with two experimental trials: exercise and control. A randomisation scheme was generated by the lead author using *GraphPad Software 2018*^©^ to allocate the order of experimental trials. The control and exercise trials were separated by a 1-week wash-out period. Before experimental trials, participants underwent screening followed by a submaximal exercise test. All aspects of the study took place in a metabolic research unit of the New Lister Building at the University of Glasgow.

### Screening

Participants completed physical activity readiness (PARQ) [24], healthy screening and international physical activity questionnaire [25]. Only participants with light or moderate physical activity levels and those who answered ‘no’ to all questions in PARQ were enroled to the study. Subsequently, height and body mass were measured, and participants conducted a submaximal exercise test.

### Submaximal exercise test

The test was conducted on a treadmill (Trackmaster Treadmills, Full Vision, Inc., Kansas, USA). After a 4-min warm-up (walking on treadmill at 3.5 km/h), participants walked on the treadmill at a constant speed of 6 km/h with the incline being increased by 2% every 4 min. The whole test consisted of 4–6 stages (16–24 min in total). The test was terminated once the participant reached 85% of their age-predicted maximal heart rate (HR_max_ = 220 – age). During the test, participants were wearing a HR monitor and a face mask, both connected to indirect calorimetry equipment (Quark RMR®, COSMED, Italy). Maximal oxygen uptake (V̇O_2_ max) was predicted by extrapolation of the HR against V̇O_2_ plot to age-predicted maximum HR [26, 27]. Data obtained during submaximal tests were used to calculate the treadmill speed and incline during the exercise trials.

### Experimental trials

Two days before the first trial, participants recorded all food consumed using a weighed food diary and replicated intake before the second trial. They also refrained from exercise and alcohol intake, but sleep was not monitored. On each trial morning, participants arrived at the laboratory between 08:00 and 09:00 after an overnight fast [28]. Height (Seca, Leicester, UK), body mass (TANITA-TBF-310, UK), and indirect calorimetry measurements were conducted. A cannula was inserted into an antecubital vein, and after 10 min, a baseline blood sample was taken, and subjective appetite was measured. Participants then either rested (control trial) or exercised (exercise trial) for 1 h. Exercise was performed on a treadmill at 60% V̇O_2_ max, an intensity sustainable for 1 h in normal weight and overweight individuals, and known to influence appetite-related measures [29]. Participants wore a heart rate monitor and a face mask connected to indirect calorimetry equipment (Quark RMR®, COSMED, Italy). Oxygen consumption (V̇O_2_) and carbon dioxide production (V̇CO_2_) were measured to calculate fat and CHO oxidation and energy expenditure [26].

One hour after exercise or rest, participants received an isocaloric meal identical in weight and macronutrient composition across trials. Meals included PKU SLPF, a protein substitute, and natural free protein foods, providing 623 kcal with 65%, 13% and 22% of energy from CHO, protein and fat, respectively (Table 1). Meals were consumed within 20 min. Appetite questionnaires and blood samples were collected every 30 min during pre-meal (0–120 min) and post-meal (120–300 min) periods. Energy expenditure was measured via indirect calorimetry for 20 min after each blood sample.

**Table 1:** Macronutrient and energy distribution of the standardised meals.

Food items	Portion size^a^	CHO (g; kcal)	Protein (g; kcal)	Fat (g; kcal)
Strawberry jam	20 g	13; 52	0	0
Promin® bread^b^	48 g	25; 100	0	2; 18
Taranis® lemon cake^b^	40 g	25; 100	0	6; 54
Lophlex® protein substitute^b^	125 ml	9; 36	20; 80	0
Slightly salted butter	4 g	0	0	3; 27
Green grapes	15 g	3; 12	0	0
Red apple	67 g	8; 32	0	0
Mevalia® strawberry bar^b^	25 g	19; 76	0	4; 36
Total (g; kcal)		**103; 408**	**20; 80**	**15; 135**
Energy (%)		**65**	**13**	**22**

Bold values reflect the total amount of CHO, protein, and fat obtained within the test meal and the proportion of energy provided by CHO, protein, and fat provided by the test meal.

*CHO* carbohydrate, *PS* protein substitute.

^a^Portion sizes of the standardised meals consumed by participants in the Exercise and the control trials.

^b^PKU type foods.

### Indirect calorimetry measurements

Measurements of V̇O_2_ and V̇CO_2_ were conducted by means of computerised open-circuit ventilated-hood system (Quark RMR®, COSMED, Italy) and used to estimate energy expenditure, fat oxidation, CHO oxidation and RER. Each measurement was carried out for ~15 min while participants were lying comfortably in the supine position. A weekly alcohol burning validation test was conducted and revealed average coefficient of variation of ~1.5%. Calculations of V̇O_2_ and V̇CO_2_ were determined by using indirect calorimetry equations described by Frayn and Macdonald [26]. Due to technical faults, metabolic rate measurements were conducted on 12 of the 16 participants.

### Appetite scores

Appetite was assessed using a validated visual analogue scale questionnaire containing 100 mm scales [30]. Participants marked a point that reflected their feelings of hunger, fullness, satiety, desire to eat and prospective food consumption (PFC). A composite appetite score (CAS), which integrates the appetite sensations into one index associated was calculated: [(hunger + PFC + desire to eat) + (100 – fullness) + (100 – satiety)] ÷ 5 [31].

### Blood sampling and analysis

Venous blood samples used for the analysis of GDF-15 were collected into ethylenediamine tetra-acetic acid (EDTA) vacuette tubes (Greiner Bio-One, Kremsmünster, Austria). Blood samples for the determination of total PYY and total GLP-1 were collected into Aprotinin EDTA tubes (400 kIU activity per ml, Sigma Aldrich, UK). After centrifugation (4 °C, 3000 rpm for 15 min), plasma was aliquoted into Eppendorf tubes for the storage at 80 °C until further analysis. ELISA kits were used to measure concentrations of GLP-1 and PYY (Merck, Millipore, Bioscience Division, UK) and plasma GDF-15 (R&D Systems, Bio-Techne, UK). Coefficients of variation were <8% for GLP-1, PYY and GDF-15 assays.

### Statistical analysis

Linear mixed models, with trial (exercise or control) and timepoint as fixed factors, were used to examine changes in outcomes. All models included a random effect for each subject. Bonferroni-adjusted pairwise comparisons were performed when significant effects were found in linear mixed models. We calculated approximate omega squared (*ω*^2^) effect size values for fixed effects to supplement linear mixed models. Further, as a summary statistic that is interpretable and relevant to our study’s aim, time-averaged values for the pre-meal (0–120 min) and post-meal (120–300 min) periods were calculated within subjects by averaging values (mean) during periods. These were then compared between trials by paired *t*-test. We also calculated absolute Hedge’s effect size values, with effect size values of <0.20, 0.20–0.49, 0.50–0.79 and ≥0.80 considered trivial, small, moderate and large, respectively [32]. Statistical analyses were performed using IBM SPSS® (version 25) and Minitab® (version 17.3.1; Minitab, Inc., State College, PA). Descriptive data are presented as mean ± SD and inferential data are presented as mean ± standard error mean, unless noted otherwise. A *P* value < 0.05 denotes statistical significance.

## Results

### Participants

The study was completed by 16 participants (Supplementary Fig. 1). The participants characteristics were: age: 26.5 ± 4.8 years, BMI: 23.7 ± 3.2 kg/m^2^ and V̇O_2_ max: 42.0 ± 7.8 ml/kg/min. Mean (±SD) body weight (control, 70.8 ± 10.3 kg; exercise, 70.7 ± 10.3 kg) and BMI (control, 23.2 ± 2.5 kg/m^2^; exercise, 23.1 ± 2.4 kg/m^2^) measured before the start of the experimental trials were not different (*P* ≥ 0.920). Between-trial baseline appetite hormones, appetite, energy expenditure, fat oxidation, CHO oxidation and RER values were also not statistically different (*P* ≥ 0.090; Supplementary Table 1).

### Responses of GLP-1 and PYY

**Fig. 1:**
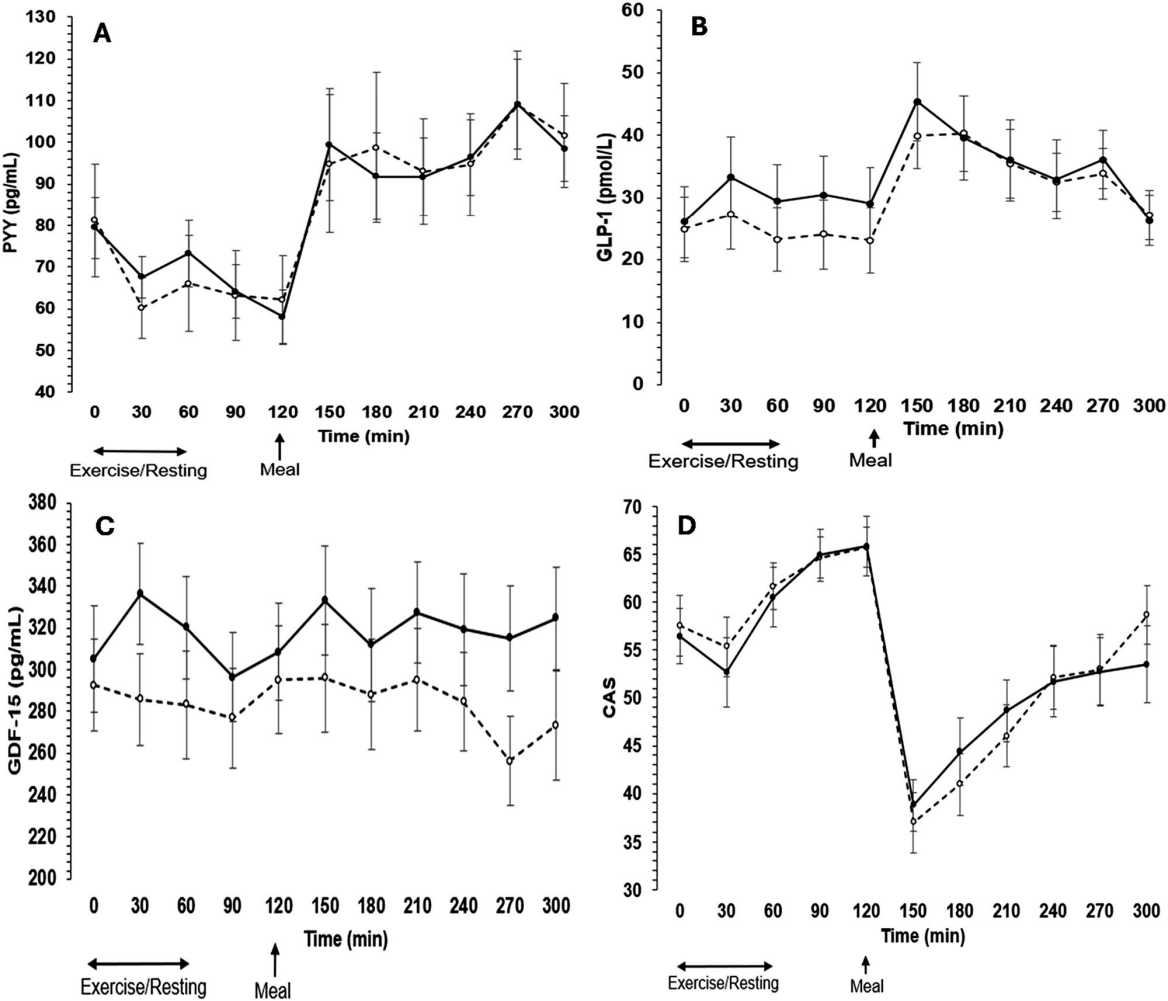
Responses of plasma gastrointestinal appetite hormones and composite appetite score during control and exercise trials. Responses of plasma concentrations of peptide YY (PYY) (**A**), glucagon like peptide (GLP-1) (**B**), growth differentiation factor-15 (GDF-15) (**C**) and the composite appetite score (CAS) (**D**) over time during the control and the exercise trials (*n* = 16). Unfilled symbols are control; black symbols are exercise. Exercise/resting period occurred at timepoints 0–60 min; meal provided at timepoint 120 min. Values are means ± SE.

Linear mixed models revealed no significant trial by time interactions for GLP-1 and PYY (*P* ≥ 0.316, *ω*^2^ ≤ 0.01; Fig. 1). However, although not evident for PYY (*P* = 0.857), a significant trial effect was shown for GLP-1, with concentrations higher in exercise than control (*P* < 0.001, *ω*^2^ = 0.04). There was also a significant main effect of time for GLP-1 and PYY due to the meal (*P* < 0.001, *ω*^2^ ≥ 0.33). Time-averaged plasma concentrations of GLP-1 were 5 (±2) pmol/L higher in the exercise trial than the control trial for the pre-meal period (*P* = 0.017), with a moderate ES show (ES = 0.65); but no differences were found during the post meal period (between-trial difference: 2 ± 2 pmol/L; *P* = 0.279, ES = 0.27; Table 2). No differences were found during the pre-meal (between-trial difference: 1 ± 7 pg/mL) and the post-meal (between-trial difference: −1 ± 7 pg/mL) periods for PYY (*P* ≥ 0.843, ES ≤ 0.05; Table 2).

**Table 2:** Time-averaged values for appetite hormones, appetite, energy expenditure, fat oxidation, CHO oxidation and RER values for pre-meal (0–120 min), post-meal (120–300 min) and the entire period (0–300 min) of the control and exercise trials.

	Control		Exercise		*P*	Effect size
Glucagon-like peptide 1 (pmol/L)						
0–120 min	24	±5	29	±6	0.017*	0.65
120–300 min	32	±5	34	±5	0.279	0.27
Peptide YY (pg/mL)						
0–120 min	68	±11	70	±6	0.843	0.05
120–300 min	90	±12	89	±8	0.881	0.04
Growth differentiation factor-15 (pg/mL)						
0–120 min	286	±23	314	±23	0.002*	1.00
120–300 min	288	±25	322	±24	0.002*	0.94
Composite appetite score						
0–120 min	62	±2	61	±3	0.667	0.11
120–300 min	50	±3	51	±3	0.855	0.05
Energy expenditure (kcal/min)						
0–120 min	1.19	±0.08	4.66	±0.24	<0.001*	4.48
120–300 min	1.39	±0.06	1.42	±0.05	0.543	0.17
Fat oxidation (g/min)						
0–120 min	0.08	±0.01	0.23	±0.02	<0.001*	1.97
120–300 min	0.09	±0.01	0.11	±0.01	0.013*	0.80
Carbohydrate oxidation (g/min)						
0–120 min	0.12	±0.02	0.76	±0.07	<0.001*	2.61
120–300 min	0.16	±0.01	0.12	±0.01	0.022*	0.71
Respiratory exchange ratio						
0–120 min	0.82	±0.01	0.84	±0.01	0.055	0.58
120–300 min	0.83	±0.01	0.79	±0.01	0.005*	0.94

*n* = 16 for glucagon-like peptide-1, peptide YY, growth differentiation factor-15, composite appetite score; *n* = 12 for energy expenditure, fat oxidation, carbohydrate oxidation, respiratory exchange ratio.

Data are mean ± SE.

*P* value determined through paired *t*-test.

*P < 0.05.

### Growth differentiation factor-15 (GDF-15)

Responses of GDF-15 were higher (*P* < 0.001, *ω*^2^ = 0.21, trial effect) in the exercise trial than the control trial, but changes over time were not significant and there was no trial by time interaction (*P* > 0.156, *ω*^2^ ≤ 0.02; Fig. 1). Compared to control, time-averaged plasma concentrations of GDF-15 were higher during the pre-meal (between-trial difference: 28 ± 7 pg/mL) and post-meal (between-trial difference: 34 ± 9 pg/mL) periods (*P* = 0.002), with large ES values for both periods (ES ≥ 0.94; Table 2).

### Subjective appetite scores

The CAS was not different between trials (*P* = 0.832, *ω*^2^ < 0.01, trial effect; Fig. 1) and there was no trial by time interaction (*P* = 0.903, *ω*^2^ < 0.01), yet there was a main effect of time due to the meal (*P* < 0.001, *ω*^2^ = 0.44, time effect; Fig. 1). Between-trial differences in time-averaged CAS were not different during both periods (*P* ≥ 0.667; ES ≤ 0.11; Table 2). Supplementary Fig. 2 shows appetite ratings for scores that make up the CAS.

### Energy expenditure and energy substrate utilisation

**Fig. 2:**
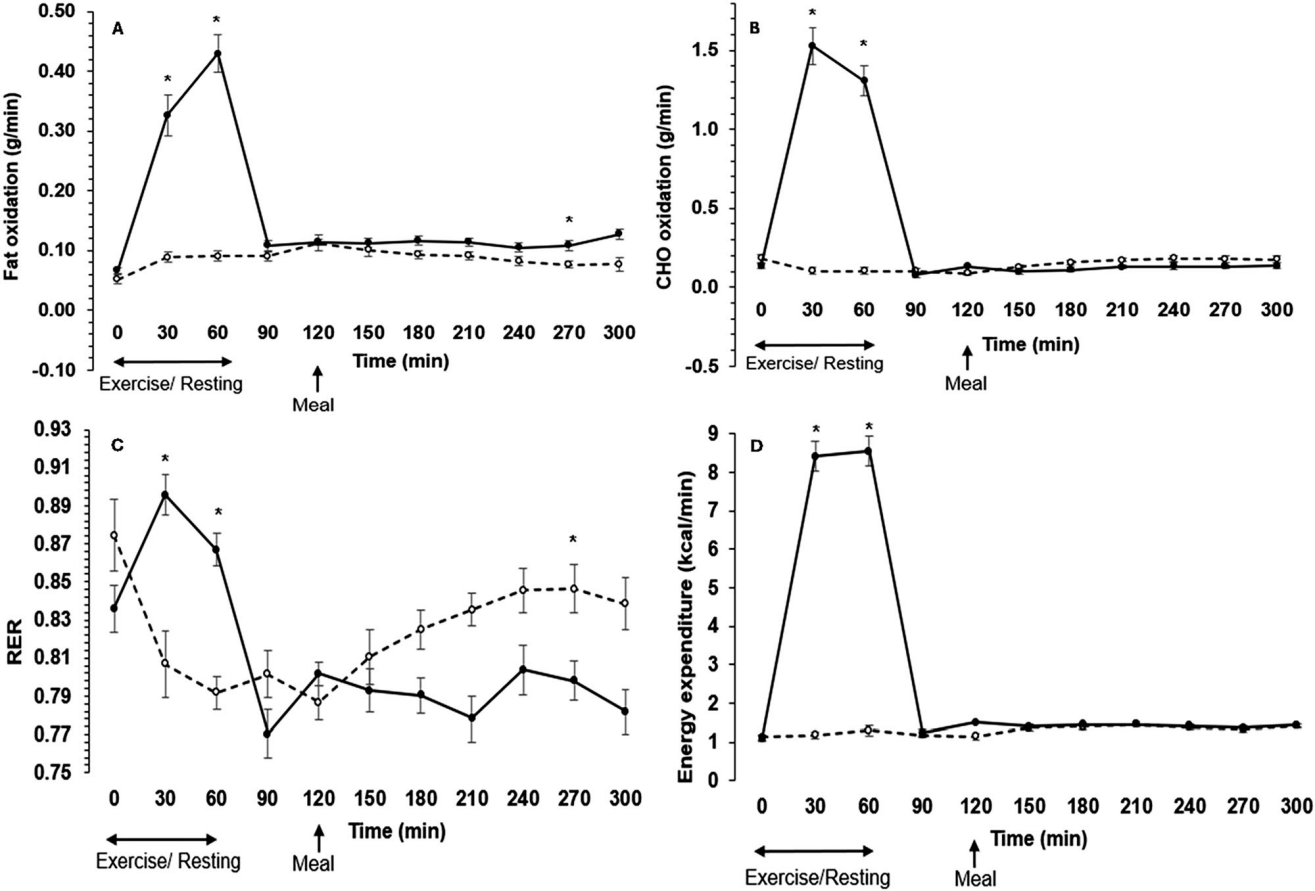
Metabolic responses during the control and exercise trials. Responses of fat oxidation (**A**), carbohydrate (CHO) oxidation (**B**), respiratory exchange ratio (RER) (**C**) and energy expenditure (**D**) over time during the control and the exercise trials (*n* = 12). Unfilled symbols are control; black symbols are exercise. Exercise/resting period occurred at timepoints 0–60 min; meal provided at timepoint 120 min. Values are means ± SE. *Different between control and exercise.

Linear mixed models for energy expenditure, fat oxidation, CHO oxidation and RER showed a main effect of time (*P* < 0.001, *ω*^2^ ≥ 0.21), further to a main effect of trial that indicated greater energy expenditure, fat oxidation and CHO oxidation, and lower RER during exercise relative to control (*P* ≤ 0.038, *ω*^2^ ≥ 0.02; Fig. 2). There was also a significant trial by time interaction effect for these outcomes (*P* < 0.001, *ω*^2^ ≥ 0.28). Post hoc comparisons revealed higher energy expenditure, fat oxidation, CHO oxidation and RER at 30 and 60 min during exercise (*P* ≤ 0.011), as well as greater and lower fat oxidation and RER, respectively, at 270 min in the postprandial state (*P* ≤ 0.036; Fig. 2). Between 0 and 120 min, average energy expenditure, fat oxidation and CHO oxidation values were greater during exercise than control (*P* < 0.001; ES ≥ 1.97), but there was a tendency for RER values to be greater during exercise in this period (*P* = 0.055; ES = 0.58; Table 2). Between 120 and 300 min, there was no difference in energy expenditure between trials (*P* = 0.543; ES = 0.17); yet compared to control, fat and CHO oxidation were higher and lower, respectively and RER was reduced in the exercise trial (*P* ≤ 0.022; ES ≥ 0.71; Table 2).

## Discussion

This study found that, in a healthy population without obesity, acute exercise of moderate intensity conducted 1 h prior to a typical PKU meal (comprised of PKU special low protein foods, a protein substitute and natural free protein foods) had no impact on postprandial responses of GLP-1 and PYY. Exercise, however, led to higher concentrations of GDF-15, did not trigger compensatory elevations in appetite, and upregulated fat oxidation. Thus, despite no changes in gut hormones, our data may imply that regular exercise sessions could favourably improve appetite regulation and body weight management in individuals consuming PKU-like meals.

Acute exercise can lead to increased PYY and GLP-1 concentrations in healthy participants [16, 17, 29]. Compared to control, we observed higher GLP-1 in the exercise trial during pre-meal period when exercise occurred, but there was no between-trial difference in postprandial state, with a trivial average difference of 2 pmol/L. We also found no differences in PYY concentrations during the fasting and postprandial state. One reason could be the relatively low intensity of the exercise (60% V̇O_2_ max), since intensities greater than or equal to 70% V̇O_2_ max generally increase satiety peptides in the postprandial state [33]. It could be that exercise greater than or equal to 70% V̇O_2_ max may be needed to stimulate elevations in PYY and GLP-1 following a PKU-like meal. Nonetheless, Douglas and colleagues found that exercise at 60% V̇O_2_ max led to higher PYY and GLP-1 in the fasting and postprandial state in individuals who are of normal weight [29]. The low protein content of the PKU-like meal may explain why exercise did not change PYY or GLP-1 post-meal, although variations in biochemical analysis could also influence variability [34]. Further work should examine the effect of acute exercise on postprandial appetite-related measures after PKU- and non-PKU meals.

Concentrations of GDF-15 were significantly higher during entire period of the study, including pre- and post-meal periods. These findings support previous studies reporting exercise-induced increases in GDF-15 in the fasted [35, 36] and postprandial states [19]. The current study is the first, to our knowledge, to assess GDF-15 alongside subjective appetite in males without obesity. That GDF-15 was higher in the postprandial state, but PYY and GLP-1 were not could be due to differences in release. Indeed, during exercise, GDF-15 is released from liver [37] and muscle [38], whilst GLP-1 and PYY are principally secreted from the gut. Further, although the exercise-induced increase in GDF-15 may be implicated in blood glucose regulation [39], it is possible that GDF-15 influences appetite differently than PYY and GLP-1 due to its primary binding to its receptor on the hindbrain [19, 20]. More work is needed to establish the mechanistic relationships between GDF-15 and appetite, as subjective appetite was unaffected by exercise and food intake was not measured.

Postprandial fat oxidation was higher after exercise, supporting other research [22, 40, 41], and we found CHO oxidation and RER were lower during the post-meal period in the exercise trial versus control. Additionally, EE was higher during the exercise trial, supporting previous work [42]; yet there were no differences postprandially. A PKU-type meal induces lower postprandial EE (or diet-induced thermogenesis) and fat oxidation [15], but the present study indicates that acute exercise may offset these reductions. Given the positive relationship between RER and energy intake (potentially mediated by the regulation of peripheral fatty acid oxidation in muscle) [11, 43], the decreased RER post-exercise may also improve appetite regulation after a meal. Moreover, exercise-induced elevations in GDF-15 may have increased fat oxidation in the current study [44, 45]. Collectively, our findings indicate acute exercise could enhance metabolic outcomes that are associated with improved appetite regulation after a PKU-type meal, although work is needed to elucidate the causal interplay between outcomes during exercise.

Despite our novel findings, work is needed on exercise and appetite responses in PKU patients. Mazzola and colleagues found similar exercise-induced metabolic responses in PKU patients and healthy controls [46], but postabsorptive or postprandial appetite, appetite-related hormones and substrate oxidation were not assessed. Work is also needed to examine the influence of exercise training on energy balance outcomes in PKU patients. Indeed, while exercise may create an acute negative energy balance in PKU patients, it is unclear if it negates reduced EE and fat oxidation from multiple PKU meals. Moreover, chronic exercise can increase energy intake in healthy participants with overweight and obesity [47], although there is heterogeneity [48, 49] and some inconsistencies [50] which could be related to energy intake assessment methods. Exercise training studies could inform physical activity guidelines for PKU patients. This is important given low activity levels in this population [51].

This study has limitations. Our study was performed in healthy males, so further research is needed in PKU patients and females. Other appetite hormones, such as ghrelin, were also not measured. Additional studies with more appetite-related agents and active isoforms (e.g. PYY_3-36_) are needed due to the array of appetite hormones and their relative redundancy [52]. No a priori power calculation was performed, so the sample may have been underpowered for some outcomes, especially for substrate oxidation and EE measures; though effect sizes for null postprandial differences were small. Finally, it is possible that additional trials would have improved the study’s conclusions: exercise and rest after non-PKU meals. Nevertheless, for pragmatic reasons, we opted to have two trials to efficiently answer our primary aim.

## Conclusion

In healthy participants without obesity, performing exercise prior to a PKU meal has no impact on postprandial GLP-1, PYY, EE and appetite. Nonetheless, exercise increases postprandial concentrations of GDF-15 and fat oxidation, and it reduces CHO oxidation and RER, potentially negating postprandial rises in appetite. Training studies are needed in PKU patients, but these findings indicate that exercise could benefit appetite regulation, thus preventing overconsumption and assisting weight management in PKU patients.

## Supplementary information

Supplementary material

## Data Availability

Data from this study are available from the corresponding author on reasonable request.
