# Supplementary material for: Effects of exercise conducted prior to phenylketonuria-type meal on appetite, satiety hormones and energy expenditure: a randomised cross-over trial
Source: Eur J Clin Nutr. 2025 May 23;79(12):1197–203. doi: 10.1038/s41430-025-01629-7 (PMC12678187; doi:10.1038/s41430-025-01629-7)
Supplement: Supplementary file 1 — Supplementary material [file 41430_2025_1629_MOESM1_ESM.docx]

**Supplementary materials**

Supplementary material for Alghamdi et al. *Effects of exercise conducted prior to phenylketonuria-type meal on appetite, satiety hormones and energy expenditure: a randomised cross-over trial.*


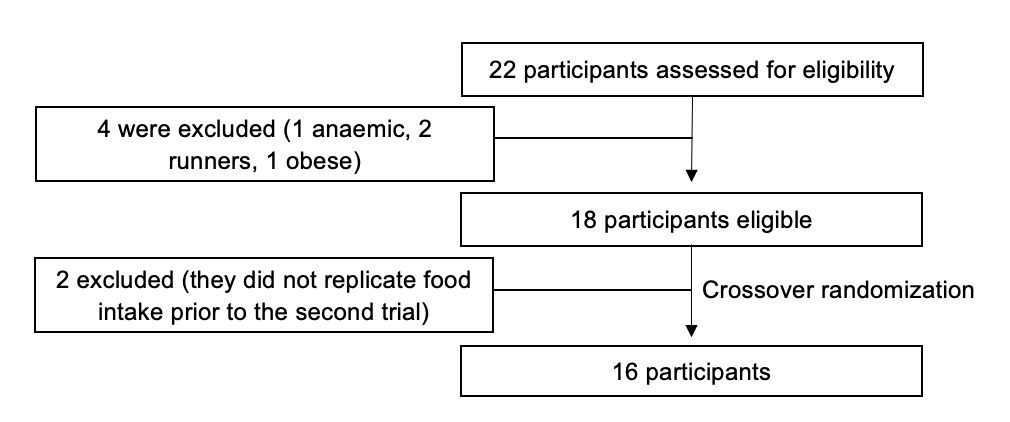


**Supplementary Figure 1.** Flowchart of participant recruitment and screening

**Supplementary Table 1.** Baseline appetite hormones, appetite, energy expenditure, fat oxidation, CHO oxidation, and RER values in the control and exercise trials

|  | **Control** | | **Exercise** | | ***P*** |
| --- | --- | --- | --- | --- | --- |
| Glucagon-like peptide 1 (pmol/L) | 25 | ± 21 | 26 | ± 23 | 0.408 |
| Peptide YY (pg/mL) | 81 | ± 51 | 79 | ± 28 | 0.834 |
| Growth differentiation factor-15 (pg/mL) | 292 | ± 88 | 318 | ± 102 | 0.141 |
| Composite appetite score | 58 | ± 12 | 56 | ± 12 | 0.759 |
| Energy expenditure (kcal/min) | 1.12 | ± 0.23 | 1.08 | ± 0.22 | 0.478 |
| Fat oxidation (g/min) | 0.05 | ± 0.03 | 0.07 | ± 0.02 | 0.190 |
| Carbohydrate oxidation (g/min) | 0.18 | ± 0.10 | 0.14 | ± 0.06 | 0.090 |
| Respiratory exchange ratio | 0.87 | ± 0.07 | 0.84 | ± 0.05 | 0.141 |

n = 16 for glucagon-like peptide-1, peptide YY, growth differentiation factor-15, composite appetite score; n = 12 for except for energy expenditure, fat oxidation, carbohydrate oxidation, respiratory exchange ratio.

Values are mean ± SD.

*P* value determined through paired t-test.


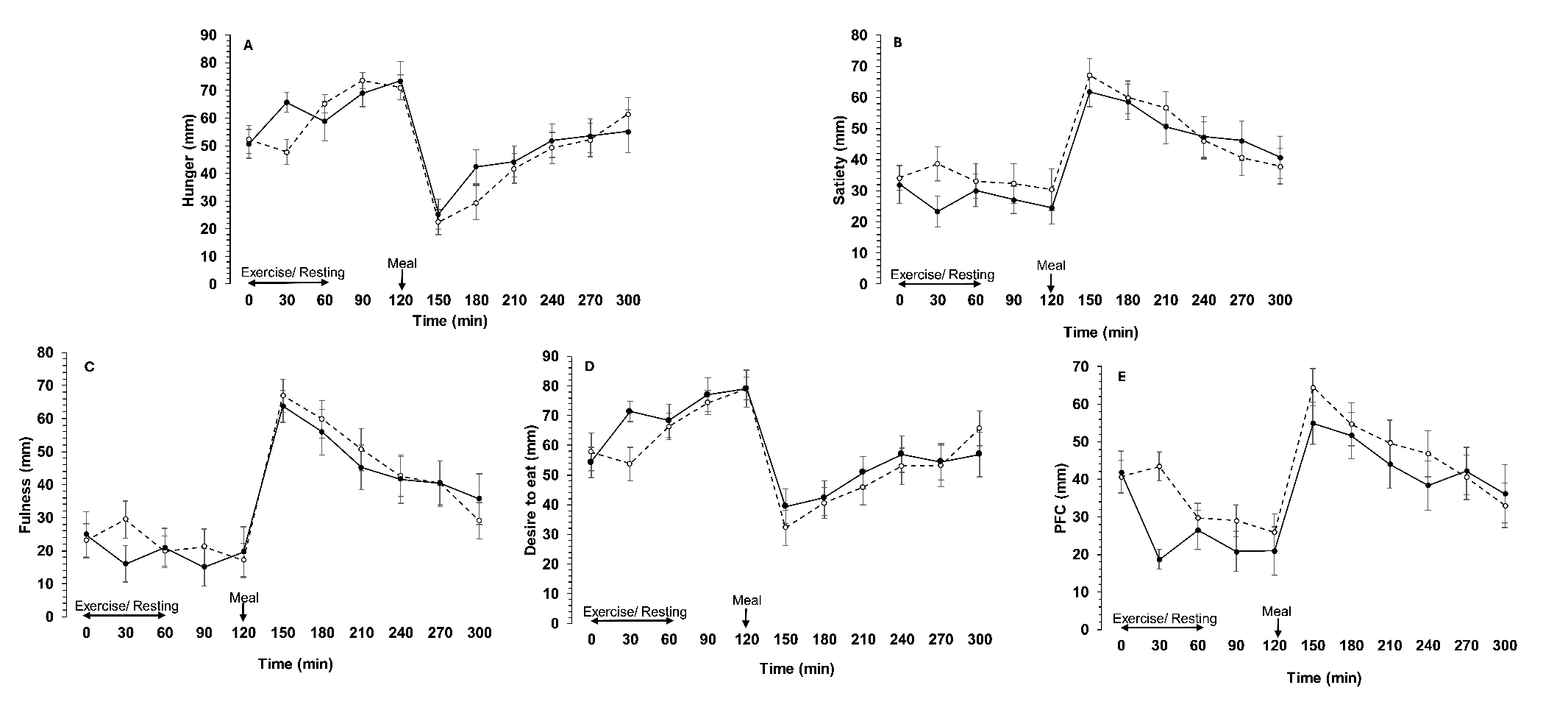


**Supplementary Figure 2.** Responses of hunger (A), satiety (B), fullness (C), desire to eat (D), and prospective food consumption (PFC) (E) over time during the control and the exercise trials (n= 16). Unfilled symbols are control; black symbols are exercise. Exercise/resting period occurred at timepoints 0-60 min; meal provided at timepoint 120 min. Values are means ± SE.
